# Supplementary material for: Rational design of universal immunotherapy for TfR1-tropic arenaviruses
Source: Nat Commun. 2020 Jan 3;11:67. doi: 10.1038/s41467-019-13924-6 (PMC6941993; doi:10.1038/s41467-019-13924-6)
Supplement: Supplementary file 1 — Supplementary Information [file 41467_2019_13924_MOESM1_ESM.pdf]

Supplementary information for:

Rational Design of Universal Immunotherapy for TfR1-tropic Arenaviruses

Hadas Cohen-Dvashi[1], Ron Amon[2], Krystle N Agans[3, 4], Robert W Cross[3, 4], Aliza Borenstein-Katz[1], Mathieu Mateo[5], Sylvain Baize[5], Vered Padler-Karavani[2], Thomas W Geisbert[3, 4], and Ron Diskin[1*]

[1]Department of Structural Biology, Weizmann Institute of Science, Rehovot 7610001, Israel

[2]Department of Cell Research and Immunology, Tel Aviv University, Tel Aviv 69978, Israel

[3]Galveston National Laboratory, University of Texas Medical Branch, Galveston, TX 77555

[4]Department of Microbiology and Immunology, University of Texas Medical Branch, Galveston, TX 77555

[5]Unité de Biologie des Infections Virales Emergentes, Institut Pasteur, Centre International de Recherche en Infectiologie (INSERM, CNRS, ENS Lyon, Université Lyon I), Lyon, France

- Corresponding author. Email: ron.diskin@weizmann.ac.il

This file contains:

Supplementary table 1

Supplementary figures 1-7

Supplementary Table 1 – Data collection and refinement statistics

**Data collection**	
Wavelength (Å)	0.9198
Space group	$P\,4_3\,2\,2$
Cell dimensions	
a, b, c (Å)	104.6 104.6 281.4
α, β, γ °	90 90 90
Resolution (Å)	50.00-2.7 (2.75-2.7) [a]
R_{pim} (%)[b]	3.8 (48.3) [a]
$CC_{1/2}$ [b]	99.8 (30.0) [a]
$I/\sigma I$ [b]	17.2 (0.9) [a]
Completeness (%)	94.3 (46.5) [a]
Multiplicity	10.2
Total reflections	425370
Unique reflections	41703
**Refinement**	
Resolution (Å)	49.56 - 2.70
No. of reflections	36590
R_{work}/R_{free} (%)	24.1 / 26.6
No. of atoms	
Protein	9542
Glycans	131
Water	42
B factors	
Protein	79.5
Glycans	76.4
Water	43.7
Ramachandran	
Favored (%)	95.5
Allowed (%)	4.3
Outlier (%)	0.2
Root mean square deviations	
Bond length (Å)	0.006
Bond angles °	0.949

[a] Values in parentheses are for the highest resolution-shell

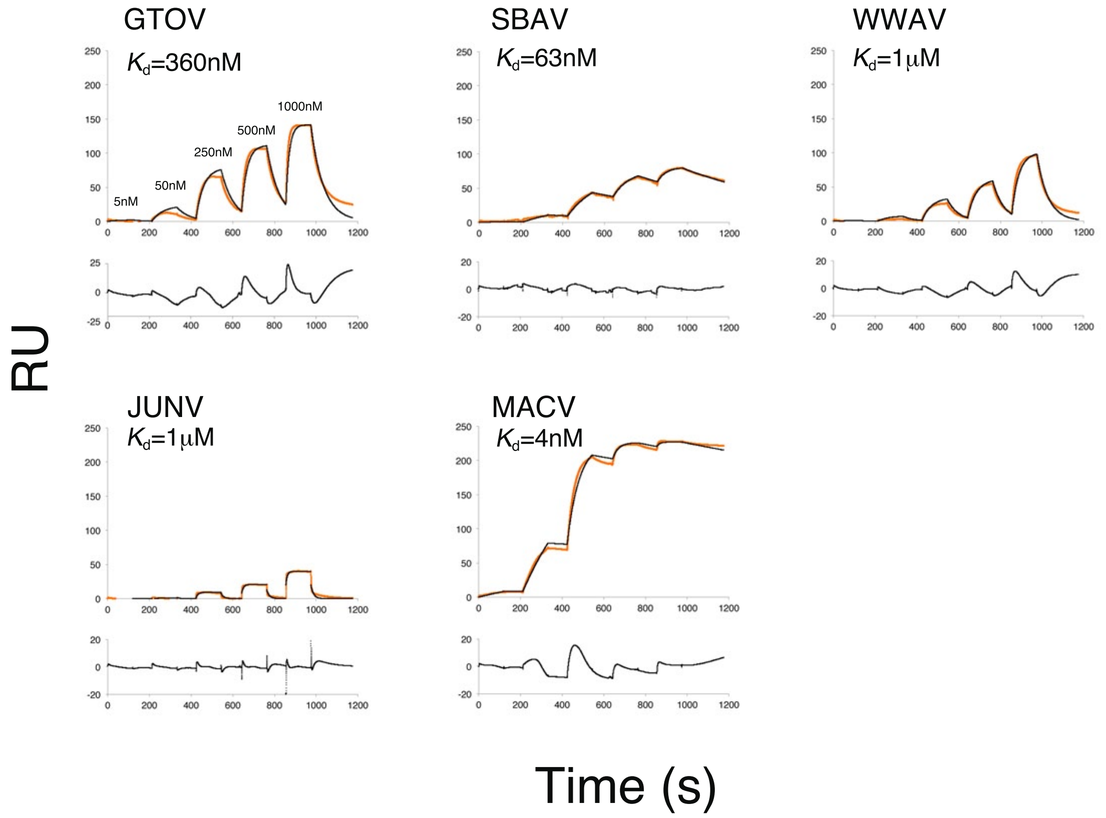

Supplementary Figure 1 | Measurements of K_d values between sAD and GP1s from TfR1-tropic viruses. GP1-Fc fusion proteins were immobilized on a protein-A coated SPR sensor chip, and sAD was injected in a series of increasing concentrations (i.e. 5, 50, 250, 500, & 1000 nM) using a single cycle kinetic scheme. Representative blank-subtracted sensorgrams are shown in orange, and 1:1 binding model that was fitted to the data are shown in black. Below each sensorgram a residual plot shows the quality of the fitted model. The calculated K_d values are shown for each GP1. Each binding experiment was repeated twice.

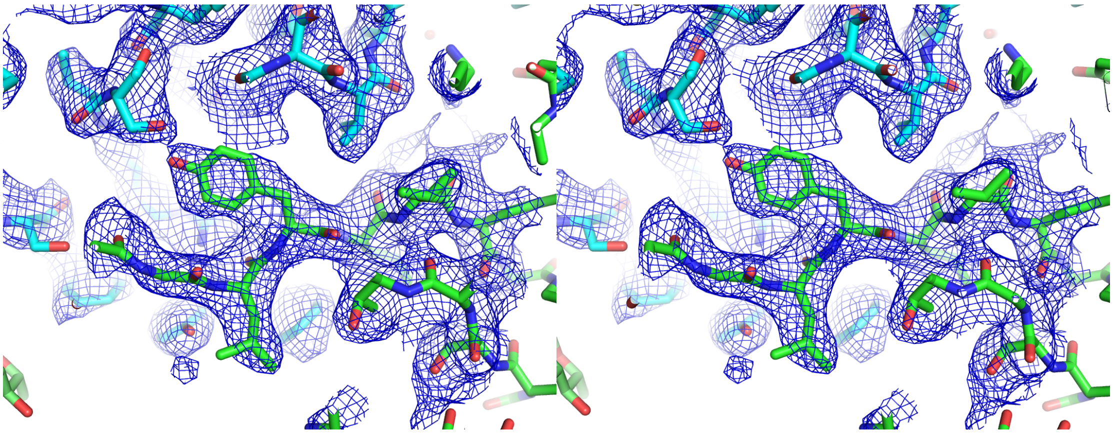

Supplementary Figure 2 | Quality of electron density. A wall-eyed stereo image of the interface between sAD (green) and $GP1_{MACV}$ (cyan) in a 2FoFc map (blue mesh) at σ=1. Clear electron density for all the side chains at the interface is visible.

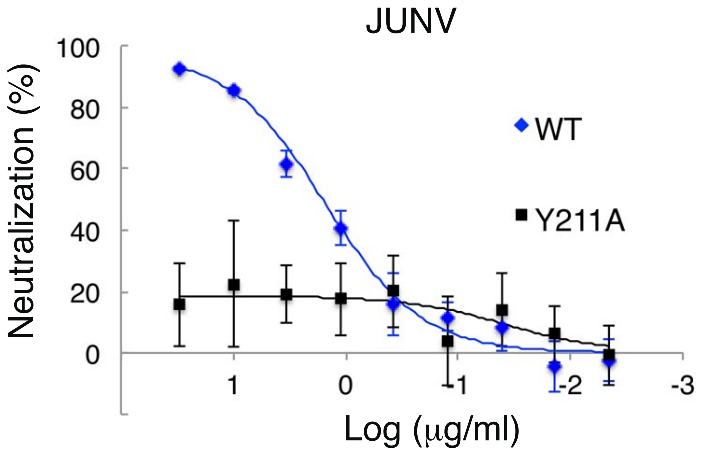

Supplementary Figure 3 | Mutating Tyr211 reduces the potency of Arenacept. A neutralization assay of pseudotyped virus bearing the spike complex of JUNV by Arenacept (blue) and an Y211A-Arenacept (black) indicating loss of potency. This is a representative graph out of three independent repeats. Error bars show standard deviations of technical repeats.

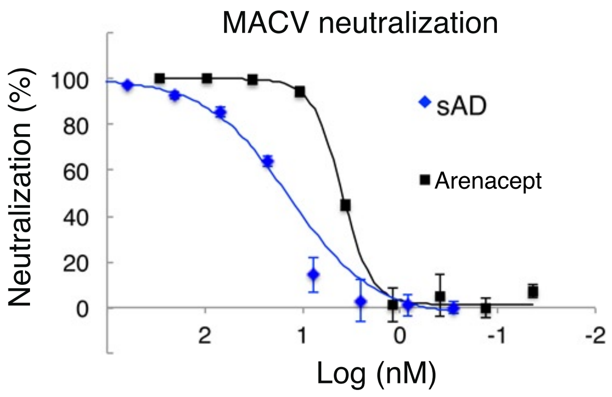

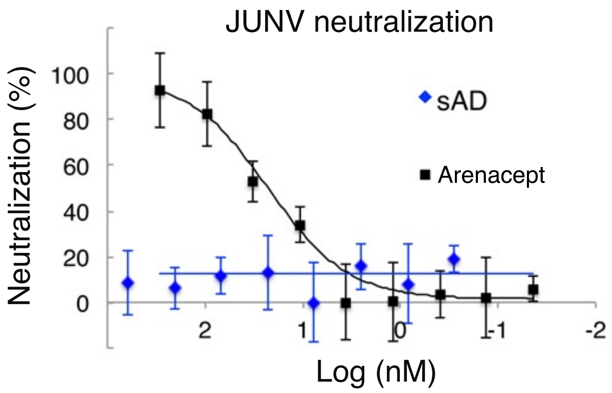

Supplementary Figure 4 | A dimeric Arenacept has higher potency compared with monomeric sAD. Neutralization assay of pseudotyped viruses bearing the spike complex of MACV or JUNV by Arenacept (black) and sAD (blue), showing elevated potency due to dimerization and indicating the effect of avidity. Since the MW of sAD and Arenacept significantly differ, the neutralization data is compared using a molarity scale. Without the effect of avidity, sAD can neutralize MACV to some degree but was practically inert toward JUNV at the range of concentrations used for this assay. These observations agree with the measured K_d values for sAD with JUNV and MACV (i.e. 1 μM and 4 nM, respectively). The neutralization curves are of representative experiments from three independent repeats. Error bars indicate standard deviations of technical repeats.

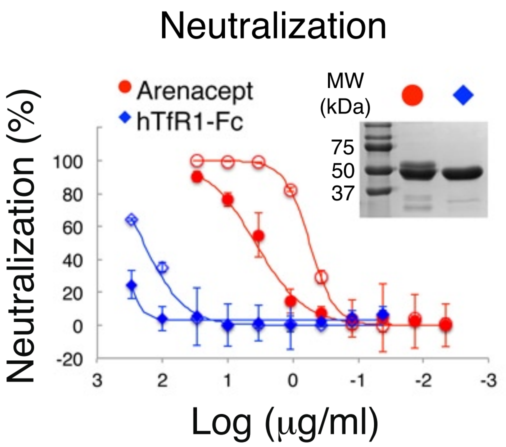

Supplementary Figure 5 | Comparison of the neutralization capacity of hTfR1-Fc vs. Arenacept. Inset shows Coomassie-stained SDS-PAGE of hTfR1-Fc (blue) and Arenacept (red). The graph shows neutralization of JUNV (closed marks) and MACV (open marks) by both Arenacept (red) and hTfR1-Fc (blue). Neutralization was determined by the reduction of reporter gene expression in the hTfR1-bearing HEK293 target cells. The neutralization curves are of representative experiments from three independent repeats. Error bars indicate standard deviations of technical repeats.

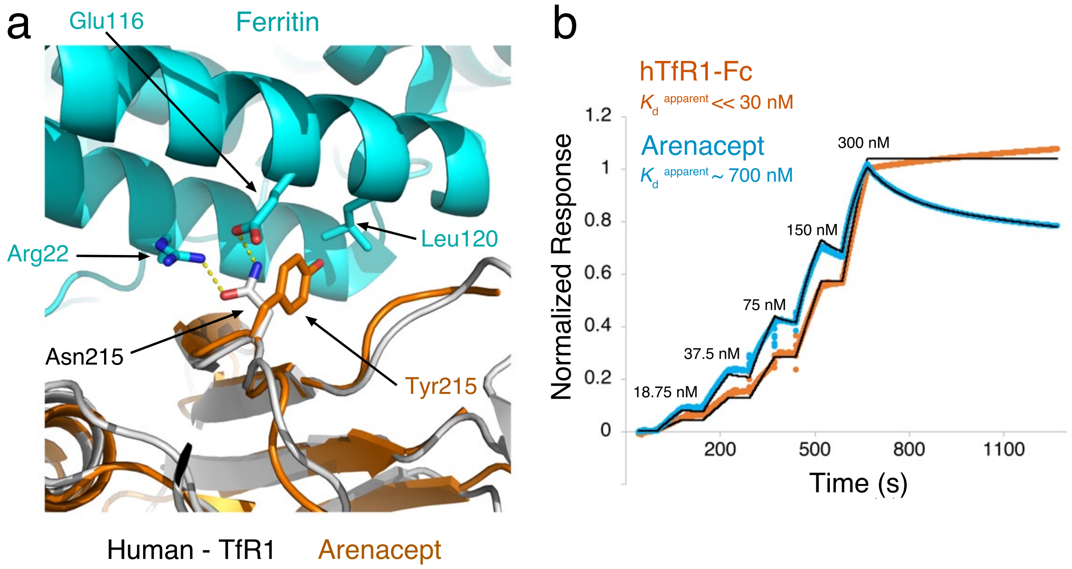

Supplementary Figure 6 | Arenacept binds to ferritin weaker than hTfR1. a. The structure of ferritin (cyan) and hTfR1 (grey) complex (PDB ID: 6H5I), superimposed with Arenacept (orange). Arg22 and Glu116 of ferritin form polar interactions with Asn215 of hTfR1. Tyr215 of Arenacept cannot form equivalent interactions and may clash with Arg22, Glu116, or Leu120 of ferritin. **b.** Normalized response of a single-cycle kinetics SPR experiment. Human ferritin was injected at the indicated concentrations over immobilized hTfR1-Fc (orange) and Arenacept (cyan). A bivalent binding model was fitted (black curves) and the calculated first K_d values are reported. Since no dissociation was observed for hTfR1-Fc, the calculated K_d can only be considered as a rough approximation of an upper boundary. This experiment was repeated twice.

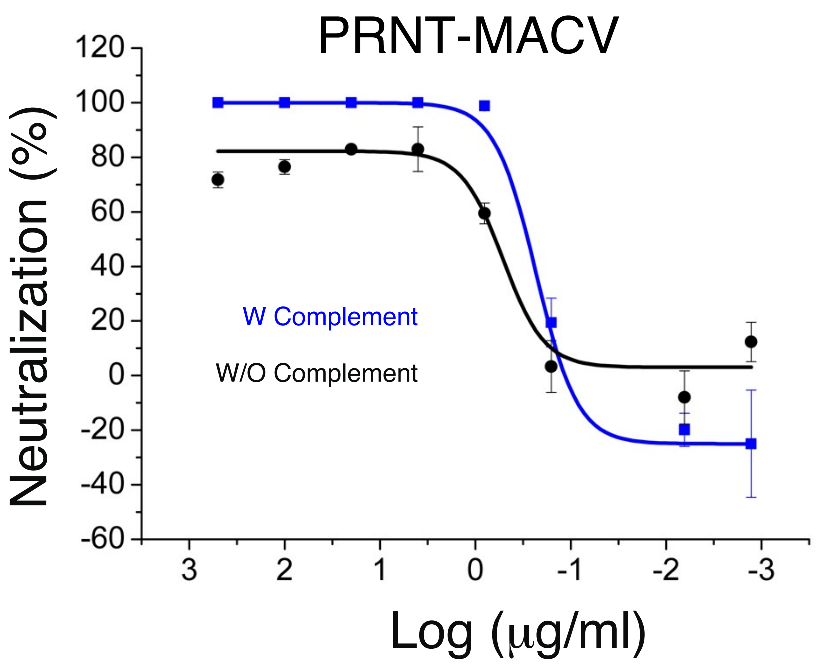

Supplementary Figure 7 | PRNT of Arenacept-M3 against MACV. Neutralization of live infectious MACV by Arenacept-M3 in the presence (blue) or absence (black) of 2.5% complement. Error bars indicate standard deviations of technical repeats.
